# New Kind of orthogonality in Normed spaces by using ϼ * -orthogonality

**DOI:** 10.12688/f1000research.174190.2

**Published:** 2026-05-27

**Authors:** Laith Ahmed Jasim - ليث احمد جاسم محمد, Mohammed Yahya Abed

**Affiliations:** 1 University of Kerbala, Karbala, Karbala Governorate, Iraq; 2 University of Kerbala, Karbala, Karbala Governorate, Iraq

**Keywords:** orthogonality, norm derivatives, ϼ_*-orthogonality.

## Abstract

Orthogonality is an important concept in normed spaces, and many generalizations of this concept have been proposed. In this paper, we introduce a new concept of orthogonality in normed spaces, denoted by $\;{ϼ}_{\mathrm{LM}}$ -orthogonality, which is related to ${ϼ}_{*}$ -orthogonality. The fundamental properties of this new concept are systematically analyzed, including symmetry, non-negativity, scalar homogeneity, linear independence of orthogonal vectors, the Cauchy–Schwarz type inequality, the zero property, and other characteristics. We also explain the relationship between this new definition and some other well-known types of orthogonality.

## 1. Introduction

In an inner product space $\left(\dot{X},\langle \cdot ,\cdot \rangle \right)$ , two vectors $\dot{s},\dot{r}\;\text{in}\;\dot{X}$ are said to be orthogonal, denoted by $\dot{s}\;⊥\;\dot{r}\;$ if and only if $\;\left\langle \dot{s},\dot{r}\;\right\rangle =0$ . In spaces whose norm is induced by an inner product, the notion of orthogonality admits a natural and uniquely determined form. Extending this concept to more general normed spaces enable a deeper investigation of their geometric and analytical interrelations, thus offering a broader framework to generalize classical Euclidean ideas to abstract mathematical settings.

Consider $\left(\dot{X},\|.\|\right)$ as a real normed vector space whose dimension is not less than two, there exist several notions of orthogonality, among which the definition introduced by (Birkhoff–James) is one of the most prominent. If $\dot{s},\dot{r}\;\in \;\dot{X}$ , define $\dot{s}\;{⊥}_{B}\;\dot{r}$ ⇔ $\left\|\dot{s}+t\dot{r}\right\|\;\geq \;\left\|\dot{s}\right\|$ *,* (t ∈R ) see. ^1^

In 1986, Amir in ^2^ defined the norm derivatives using two functionals, $\;{ϼ}_{-},{ϼ}_{+}:\dot{X}\times \dot{X}\;\to \;\mathbb{R}$ be functionals, as follows:

$$\;{ϼ}_{-}\left(\dot{s},\dot{r}\right)=\left\|\dot{s}\right\|\;\lim_{t\;\to \;0^{-}}\frac{\left\|\dot{s}+t\dot{r}\right\|-\left\|\dot{s}\right\|}{t}\;\text{and}\;\;{ϼ}_{+}\left(\dot{s},\dot{r}\right)=\left\|\dot{s}\right\|\;\lim_{t\;\to \;0^{+}}\frac{\left\|\dot{s}+t\dot{r}\right\|-\left\|\dot{s}\right\|}{t}\;$$

then ṡ is $\;{ϼ}_{-}$ - orthogonality to $\dot{r}$ denoted by $\dot{s}\;{⊥}_{{ϼ}_{-}}\dot{r}\;⟺$ $\;{ϼ}_{-}\left(\dot{s},\dot{r}\right)=0$ and ṡ is $\;{ϼ}_{+}$ - orthogonality to $\dot{r}$ denoted by $\dot{s}\;{⊥}_{{ϼ}_{+}}\dot{r}\;⟺$ $\;{ϼ}_{+}\left(\dot{s},\dot{r}\right)=0$ .

A mapping ${\left\langle \cdot ,\cdot \right\rangle }_{g}:\dot{X}\times \dot{X}\;\to \;\mathbb{R}$ was introduced by MiliĆiĆ in ^3^ as follows: $\;ϼ\left(\dot{s},\dot{r}\right)={\left\langle \dot{s},\dot{r}\right\rangle }_{g}=\frac{\;\;{ϼ}_{-}\left(\dot{s},\dot{r}\right)+{ϼ}_{+}\left(\dot{s},\dot{r}\right)\;}{2}\;$ . But M. Nur and H. Gunawan in ^4^ defined an orthogonality relation based on norm derivatives by $\;{ϼ}_{\mathrm{gg}}=\sqrt{\left|\;ϼ\left(\dot{s},\dot{r}\right)\right|\;\left|\;ϼ\left(\dot{r},\dot{s}\right)\right|}$ , Also Zamani and Moslehian in ^5^ defined an orthogonality functional $\;{ϼ}_{\lambda }::\dot{X}\times \dot{X}\;\to \;\mathbb{R}$ by $\;{ϼ}_{\lambda }$ (ṡ, $\dot{r}$ ) = λ $\;{ϼ}_{-}\left(\dot{s},\dot{r}\right)$ + (1- λ) $\;{ϼ}_{+}\left(\dot{s},\dot{r}\right)$ ∀ λ ∈ R, and ṡ is $\;{ϼ}_{\lambda }$ - orthogonality to $\dot{r}$ denoted by $\dot{s}\;{⊥}_{{ϼ}_{\lambda }}\dot{r}\;⟺$ $\;{ϼ}_{\lambda }\left(\dot{s},\dot{r}\right)=0$ the notions of $\;{ϼ}_{*}$ -orthogonality is introduced in ^6^ as: $\dot{s}\;{⊥}_{{ϼ}_{*}}\dot{r}\;⟺$ $\;{ϼ}_{*}\left(\dot{s},\dot{r}\right)={ϼ}_{+}\left(\dot{s},\dot{r}\right)\;ϼ\_\left(\dot{s},\dot{r}\right)=$ 0. Recall that $\dot{X}$ is smooth at $\dot{s}\;$ if and only if $ϼ\_\left(\dot{s},\dot{r}\right)={ϼ}_{+}\left(\dot{s},\dot{r}\right)\;$ for all $\dot{r}\;\in \;\dot{X}.$

These functionals extend the concept of the inner product to a normed space, and many geometric properties will be reformulated in the normed space using the concept of the norm derivative. Now we shall address some important theorem that we will need in this research.

Theorem 1.1: ^6^Suppose ( $\dot{X}$ , ‖·‖) represents a real normed vector space, then

(i)
$\;{ϼ}_{*}$ (t ṡ, $\dot{r}$ ) = $\;{ϼ}_{*}$ (ṡ, t $\dot{r}$ )= $t^{2}\;{ϼ}_{*}$ (ṡ, $\dot{r}$ ) for all ṡ, $\dot{r}$ ∈ $\dot{X}$ and all t ∈ R.
(ii) | $\;{ϼ}_{*}\left(\dot{s},\dot{r}\right)$ | $\;\leq \;{\left\|\dot{s}\right\|}^{2}{\left\|\dot{r}\right\|}^{2}$ for all ṡ, $\dot{r}\;\in \;\dot{X}$ .
(iii) For all vectors except the zero vector, ṡ, $\dot{r}$ ∈ $\dot{X}$ , if $\dot{s}$ $\;{⊥}_{{ϼ}_{*}}$ $\dot{r}$ , then ṡ and $\dot{r}$ are independent in the linear sense.
(iv)
$\;{ϼ}_{*}$ (ṡ, t ṡ + $\dot{r}$ ) = $t^{2}{\left\|\dot{s}\right\|}^{4}$ +2t ${\left\|\dot{s}\right\|}^{2}ϼ($ ṡ, $\dot{r}$ ) + $\;{ϼ}_{*}$ (ṡ, $\dot{r}$ ) for all ṡ, $\dot{r}\;\in \;\dot{X}$ and all t ∈ R.

For more information about other orthogonalities you can see. ^7– 10^

## 2. Fundamental results

This section is devoted to introducing a new form of orthogonality by using $\;{ϼ}_{*}$ -orthogonality as follows:

Definition 2.1: Suppose ( $\dot{X}$ , ‖·‖) represents a real normed vector space then $\forall \dot{s},\dot{r}\;\in \;\dot{X}$ , the functional ${ϼ}_{\mathrm{LM}}:\dot{X}\times \dot{X}\;\to \;R\;$ which defines as ${ϼ}_{\mathrm{LM}}\left(\dot{s},\dot{r}\right)=\sqrt{\left|{ϼ}_{*}\;\left(\dot{s},\dot{r}\right)\;{ϼ}_{*}\left(\dot{r},\dot{s}\right)\right|}$ , We say that ṡ is ${ϼ}_{\mathrm{LM}}$ - orthogonality to $\dot{r}$ , we denoted by ṡ $\;{⊥}_{{ϼ}_{\mathrm{LM}}}$ $\dot{r}$ if $\;{ϼ}_{\mathrm{LM}}\left(\dot{s},\dot{r}\right)=0$ .

The notion of ${ϼ}_{\mathrm{LM}}$ -orthogonality is motivated by the need for a symmetric orthogonality relation built upon ${ϼ}_{*}$ -orthogonality. Since ${ϼ}_{*}$ -orthogonality is not symmetric in general, we introduce a symmetric and non-negative functional that retains the core geometric structure of the original relation, thereby providing a more balanced framework for comparing vectors in normed spaces.

Proposition 2.2: Suppose ( $\dot{X}$ , ‖·‖) represents a real normed vector space. Then

(a)
${ϼ}_{\mathrm{LM}}\left(\dot{s},\dot{r}\right)\;\geq \;0$ for all ṡ, $\dot{r}\;\in \;\dot{X}$ . (non-negative)
(b)
${ϼ}_{\mathrm{LM}}\left(\dot{s},0\right)={ϼ}_{\mathrm{LM}}\left(0,\dot{r}\right)=0\;$ for all ṡ, $\dot{r}\;\in \;\dot{X}.$ (zero property)
(c)
${ϼ}_{\mathrm{LM}}\left(\dot{s},\dot{s}\right)={\left\|\dot{s}\right\|}^{4}$ for all ṡ $\;\in \;\dot{X}$ , also ${ϼ}_{\mathrm{LM}}\left(\dot{s},\dot{s}\right)$ = $\;{ϼ}_{*}\;\left(\dot{s},\dot{s}\right)$

(d)
${ϼ}_{\mathrm{LM}}\left(\dot{s},\dot{r}\right)={ϼ}_{\mathrm{LM}}\left(\dot{r},\dot{s}\right)$ for all ṡ, $\dot{r}\;\in \;\dot{X}.$ (symmetry)
(e)
${ϼ}_{\mathrm{LM}}\left(\alpha \dot{s},\beta \dot{r}\right)={\left|\alpha \beta \right|}^{2}\;{ϼ}_{\mathrm{LM}}\left(\dot{s},\dot{r}\right)$ for all $\dot{s},\dot{r}\;\in \;\dot{X}\;$ and all α,β ∈R. (scalar homogeneity)
(f )
${ϼ}_{\mathrm{LM}}\left(\dot{s},\dot{r}\right)\;\leq \;{\left\|\dot{s}\right\|}^{2}{\left\|\dot{r}\right\|}^{2}$ for all ṡ, $\dot{r}\;\in \;\dot{X}.$ (Cauchy schwarz inequality)
(g)
${ϼ}_{\mathrm{LM}}\left(\dot{s},t\dot{s}+\dot{r}\right)=$ $\sqrt{\left|\left(\;t^{2}{\left\|\dot{s}\right\|}^{4}+2t{\left\|\dot{s}\right\|}^{2}ϼ\left(\dot{s},\dot{r}\right)+{ϼ}_{*}\left(\dot{s},\dot{r}\right)\right)\;\left({ϼ}_{*}\left(t\dot{s}+\dot{r},\dot{s}\right)\right)\right|}$

(h) For all nonzero vectors ṡ, $\dot{r}\;\in \;\dot{X}\;$ , if $\dot{s}\;{⊥}_{{ϼ}_{\mathrm{LM}}}\dot{r}$ , then ṡ and $\dot{r}$ are linearly independent. **(**linear independence **)**

Proof:

(a) We have ${ϼ}_{\mathrm{LM}}\left(\dot{s},\dot{r}\right)$ = $\sqrt{\left|\;{ϼ}_{*}\;\left(\dot{s},\dot{r}\right)\;\;{ϼ}_{*}\;\left(\dot{r},\dot{s}\right)\right|}\;$ since $\left|\;{ϼ}_{*}\;\left(\dot{s},\dot{r}\right)\;\;{ϼ}_{*}\;\left(\dot{r},\dot{s}\right)\right|\;\geq \;$ 0 then $\;\sqrt{\left|\;{ϼ}_{*}\;\left(\dot{s},\dot{r}\right)\;\;{ϼ}_{*}\;\left(\dot{r},\dot{s}\right)\right|}$ ≥ 0, implies ${ϼ}_{\mathrm{LM}}\left(\dot{s},\dot{r}\right)\;\geq \;$ 0.
(b) From definition of ${ϼ}_{\mathrm{LM}}$ -orthogonality we have $\;{ϼ}_{\mathrm{LM}}\left(\dot{s},0\right)$ = $\sqrt{\left|{ϼ}_{*}\left(\dot{s},0\right)\;{ϼ}_{*}(0,\dot{s})\right|}$ = $\sqrt{\left|0.0\right|}$ = 0
  In the same way, we prove ${ϼ}_{\mathrm{LM}}\left(0,\dot{r}\right)=0$ .
(c) From definition of $\;{ϼ}_{*}$ -orthogonality and ${ϼ}_{\mathrm{LM}}$ -orthogonality we get $\;{ϼ}_{\mathrm{LM}}\left(\dot{s},\dot{s}\right)$ = $\sqrt{\left|{ϼ}_{*}\left(\dot{s},\dot{s}\right)\;{ϼ}_{*}(\dot{s},\dot{s})\right|}\;$

$$=\sqrt{\left|{ϼ}_{-}\left(\dot{s},\dot{s}\right){ϼ}_{+}(\dot{s},\dot{s})\;{ϼ}_{-}(\dot{s},\dot{s}){ϼ}_{+}(\dot{s},\dot{s})\right|}=\sqrt{\left|\;{\left\|\dot{s}\right\|}^{2}\;{\left\|\dot{s}\right\|}^{2}\;{\left\|\dot{s}\right\|}^{2}\;{\left\|\dot{s}\right\|}^{2}\;\right|}={\left\|\dot{s}\right\|}^{4}\;$$

(d)
${ϼ}_{\mathrm{LM}}\left(\dot{s},\dot{r}\right)$ = $\sqrt{\left|{ϼ}_{*}\left(\dot{s},\dot{r}\right)\;{ϼ}_{*}(\dot{r},\dot{s})\right|}\;$ = $\sqrt{\left|{ϼ}_{*}\left(\dot{r},\dot{s}\right)\;{ϼ}_{*}(\dot{s},\dot{r})\right|}\;$ = ${ϼ}_{\mathrm{LM}}\left(\dot{r},\dot{s}\right)$

(e) By theorem 1.1 (i) we have $\;{ϼ}_{*}\left(t\dot{s},\dot{r}\right)$ = ${ϼ}_{*}\left(\dot{s},t\dot{r}\right)$ = $t^{2}{ϼ}_{*}\left(\dot{s},\dot{r}\right)\;$ for all $\dot{s},\dot{r}\;\in \;\dot{X}$ and all t∈R .

${ϼ}_{*}\left(\alpha \dot{s},\beta \dot{r}\right)$ = α ^2^β ^2^ ${ϼ}_{*}\left(\dot{s},\dot{r}\right)\;$ and ${ϼ}_{*}\left(\beta \dot{r},\alpha \dot{s}\right)$ = α ^2^β ^2^ ${ϼ}_{*}\left(\dot{r},\dot{s}\right)$ then
$$\begin{array}{c} {ϼ}_{\mathrm{LM}}\left(\alpha \dot{s},\beta \dot{r}\right)=\sqrt{\left|{ϼ}_{*}\left(\alpha \dot{s},\beta \dot{r}\right)\;{ϼ}_{*}\left(\beta \dot{r},\alpha \dot{s}\right)\right|}=\sqrt{\left|\alpha^{2}\beta^{2}\;{ϼ}_{*}\left(\dot{s},\dot{r}\right)\;\alpha^{2}\beta^{2}\;{ϼ}_{*}\left(\dot{r},\dot{s}\right)\right|}\;\\ ={\left|\alpha \beta \right|}^{2}\sqrt{\left|\;{ϼ}_{*}\left(\dot{s},\dot{r}\right){ϼ}_{*}\left(\dot{r},\dot{s}\right)\right|}={\left|\alpha \beta \right|}^{2}\;{ϼ}_{\mathrm{LM}}\left(\dot{s},\dot{r}\right) \end{array}$$
(f ) By theorem 1.1 (ii) we have | ${ϼ}_{*}\left(\dot{s},\dot{r}\right)$ )| $\;\leq \;{\left\|\dot{s}\right\|}^{2}{\left\|\dot{r}\right\|}^{2}$ for all $\dot{s},\dot{r}\;\in \;\dot{X}$

  Then ${ϼ}_{\mathrm{LM}}\left(\dot{s},\dot{r}\right)=\sqrt{\left|{ϼ}_{*}\left(\dot{s},\dot{r}\right)\;{ϼ}_{*}(\dot{r},\dot{s})\right|}$ $\;\leq \;\sqrt{{\left\|\dot{s}\right\|}^{2}{\left\|\dot{r}\right\|}^{2}\;{\left\|\dot{r}\right\|}^{2}{\left\|\dot{s}\right\|}^{2}}\;$ = ${\left\|\dot{s}\right\|}^{2}{\left\|\dot{r}\right\|}^{2}$ for all $\dot{s},\dot{r}\;\in \;\dot{X}$

  Hence ${ϼ}_{\mathrm{LM}}\left(\dot{s},\dot{r}\right)\;\leq \;{\left\|\dot{s}\right\|}^{2}{\left\|\dot{r}\right\|}^{2}$ for all ṡ, $\dot{r}\;\in \;\dot{X}$

(g) By theorem 1.1 (iv) we have ${ϼ}_{*}\left(\dot{s},t\dot{s}+\dot{r}\right)$ = $t^{2}{\left\|\dot{s}\right\|}^{4}$ +2t ${\left\|\dot{s}\right\|}^{2}ϼ\left(\dot{s},\dot{r}\right)$ + ${ϼ}_{*}\left(\dot{s},\dot{r}\right)$
$${ϼ}_{\mathrm{LM}}\left(\dot{s},t\dot{s}+\dot{r}\right)=\sqrt{\left|{ϼ}_{*}\left(\dot{s},t\dot{s}+\dot{r}\right)\;{ϼ}_{*}(t\dot{s}+\dot{r},\dot{s})\right|}=\sqrt{\left|\left(\;t^{2}{\left\|\dot{s}\right\|}^{4}+2t{\left\|\dot{s}\right\|}^{2}ϼ\left(\dot{s},\dot{r}\right)+{ϼ}_{*}\left(\dot{s},\dot{r}\right)\right)\left({ϼ}_{*}\left(t\dot{s}+\dot{r},\dot{s}\right)\right)\right|}$$
(h) we have ṡ $\;{⊥}_{{ϼ}_{\mathrm{LM}}}$ $\dot{r}$ therefore; ${ϼ}_{\mathrm{LM}}\left(\dot{s},\dot{r}\right)=\sqrt{\left|{ϼ}_{*}\left(\dot{s},\dot{r}\right)\;{ϼ}_{*}(\dot{r},\dot{s})\right|}$ = 0

Thus ${ϼ}_{*}\left(\dot{s},\dot{r}\right)$ = 0 or $\;{ϼ}_{*}\left(\dot{r},\dot{s}\right)$ = 0

By theorem 1.1 (iii) for all nonzero vectors ṡ, $\dot{r}\;\in \;\dot{X}$ , if $\dot{s}$ $\;{⊥}_{{ϼ}_{*}}$ $\dot{r}$ , then ṡ and $\dot{r}$ are linearly independent.

Hence, whether ${ϼ}_{*}\left(\dot{s},\dot{r}\right)$ = 0 or $\;{ϼ}_{*}\left(\dot{r},\dot{s}\right)$ = 0 it follows that ṡ and $\dot{r}$ are linearly independent.

Remark 2.3: If $\dot{X}$ come from inner product space then ${ϼ}_{\mathrm{LM}}\left(\dot{s},\dot{r}\right)$ = $\;{ϼ}_{*}\;\left(\dot{s},\dot{r}\right)$ .

## 3. The connection between ${ϼ}_{\mathrm{LM}}$ – orthogonality and some others orthogonalities

Theorem 3.1: Suppose $\left(\dot{X},\|\cdot \|\right)$ represents a real normed vector space and let $\dot{s}$ , $\dot{r}$ ∈ $\dot{X}$ .

(a) If $\dot{s}\;\;{⊥}_{{ϼ}_{*}}\;\dot{r}$ , then $\dot{s}\;\;{⊥}_{{ϼ}_{\mathrm{LM}}}\;\dot{r}$

(b) If $\dot{s}\;\;{⊥}_{{ϼ}_{-}}\;\dot{r}$ , then $\dot{s}\;\;{⊥}_{{ϼ}_{\mathrm{LM}}}\;\dot{r}$

(c) If $\dot{s}\;\;{⊥}_{{ϼ}_{+}}\;\dot{r}$ , then $\dot{s}\;\;{⊥}_{{ϼ}_{\mathrm{LM}}}\;\dot{r}$

(d) If $\dot{r}\;{⊥}_{{ϼ}_{*}}\dot{s}$ , then $\dot{s}\;{⊥}_{{ϼ}_{\mathrm{LM}}}\dot{r}$

(e) If $\dot{r}\;{⊥}_{{ϼ}_{-}}\dot{s}$ , then $\dot{s}\;{⊥}_{{ϼ}_{\mathrm{LM}}}\dot{r}$

(f ) If $\dot{r}\;{⊥}_{{ϼ}_{+}}\dot{s}$ , then $\dot{s}\;{⊥}_{{ϼ}_{\mathrm{LM}}}\dot{r}$

Proof: Obvious

Remark 3.2: The converse of (a), (b) and (c) of theorem 3.1 are not true. For example, take $\;\dot{s}=\left(0,2\right)$ and $\dot{r}=\left(1,1\right)$ if $\|\;\left({\dot{s}}_{1},{\dot{s}}_{2}\right)\|=\max\left\{|{\dot{s}}_{1}\;|,|{\dot{s}}_{2}\;|\right\}\;$ in the space $\dot{X}$ = $R^{2}$

$$\begin{array}{c} {ϼ}_{+}\left(\dot{s},\dot{r}\right)=\left\|\dot{s}\right\|\;\lim_{t\;\to \;0^{+}}\frac{\left\|\dot{s}+t\dot{r}\right\|-\left\|\dot{s}\right\|}{t}=\left(2\right)\;\lim_{t\;\to \;0^{+}}\frac{\left|2+t\right|-2}{t}=2\;\left(1\right)=2\neq 0\\ {ϼ}_{-}\left(\dot{s},\dot{r}\right)=\left\|\dot{s}\right\|\;\lim_{t\;\to \;0^{-}}\frac{\left\|\dot{s}+t\dot{r}\right\|-\left\|\dot{s}\right\|}{t}=\left(2\right)\lim_{t\;\to \;0^{-}}\frac{\left|2+t\right|-2}{t}=2\;\left(1\right)=2\neq 0\\ \begin{array}{c} {ϼ}_{+}\left(\dot{r},\dot{s}\right)=\left\|\dot{r}\right\|\;\lim_{t\;\to \;0^{+}}\frac{\left\|\dot{r}+t\dot{s}\right\|-\left\|\dot{r}\right\|}{t}=\left(1\right)\;\lim_{t\;\to \;0^{+}}\frac{\left|1+2t\right|-1}{t}=1\left(2\right)=2\\ {ϼ}_{-}\left(\dot{r},\dot{s}\right)=\left\|\dot{r}\right\|\;\lim_{t\;\to \;0^{-}}\frac{\left\|\dot{r}+t\dot{s}\right\|-\left\|\dot{r}\right\|}{t}=\left(1\right)\;\lim_{t\;\to \;0^{-}}\frac{\left|1\right|-1}{t}= \end{array} \end{array}$$

Since $\;{ϼ}_{*}\left(\dot{s},\dot{r}\right)={ϼ}_{+}\left(\dot{s},\dot{r}\right)\;ϼ\_\left(\dot{s},\dot{r}\right)$

$$\;{ϼ}_{*}\left(\dot{s},\dot{r}\right)=\left(2\right)\left(2\right)=4\neq 0\;$$

But ${ϼ}_{\mathrm{LM}}\left(\dot{s},\dot{r}\right)=\sqrt{\left|{ϼ}_{*}\;\left(\dot{s},\dot{r}\right)\;{ϼ}_{*}\left(\dot{r},\dot{s}\right)\right|}$ = 0

Hence ṡ $\;{⊥}_{{ϼ}_{\mathrm{LM}}}\;\dot{r}\;$ but the others are not.

Remark 3.3: The converse of (d), (e) and (f ) of theorem 3.1 are not true. For example, take ṡ = (2,0,0) and $\dot{r}$ = (1,1,0) if $\left\|\;\left({\dot{s}}_{1},{\dot{s}}_{2},{\dot{s}}_{3}\right)\right\|$ = $\left|{\dot{s}}_{1}\right|+\left|{\dot{s}}_{2}\right|$ + $\left|{\dot{s}}_{3}\right|$ in the space X = $R^{3}$ , then

$$\begin{array}{c} {ϼ}_{+}\left(\dot{s},\dot{r}\right)=\left\|\dot{s}\right\|\;\lim_{t\;\to \;0^{+}}\frac{\left\|\dot{s}+t\dot{r}\right\|-\left\|\dot{s}\right\|}{t}=\left(2\right)\;\lim_{t\;\to \;0^{+}}\frac{\left|2+t\right|+\left|t\right|-2}{t}=2\left(2\right)=4\\ {ϼ}_{-}\left(\dot{s},\dot{r}\right)=\left\|\dot{s}\right\|\;\lim_{t\;\to \;0^{-}}\frac{\left\|\dot{s}+t\dot{r}\right\|-\left\|\dot{s}\right\|}{t}=\left(2\right)\lim_{t\;\to \;0^{-}}\frac{\left|2+t\right|+\left|t\right|-2}{t}=2\left(0\right)=0\\ \begin{array}{c} {ϼ}_{+}\left(\dot{r},\dot{s}\right)=\left\|\dot{r}\right\|\;\lim_{t\;\to \;0^{+}}\frac{\left\|\dot{r}+t\dot{s}\right\|-\left\|\dot{r}\right\|}{t}=\left(2\right)\;\lim_{t\;\to \;0^{+}}\frac{\left|1+2t\right|+\left|1\right|-2}{t}=2\left(2\right)=4\neq 0\\ {ϼ}_{-}\left(\dot{r},\dot{s}\right)=\left\|\dot{r}\right\|\;\lim_{t\;\to \;0^{-}}\frac{\left\|\dot{r}+t\dot{s}\right\|-\left\|\dot{r}\right\|}{t}=\left(2\right)\;\lim_{t\;\to \;0^{-}}\frac{\left|1+2t\right|+\left|1\right|-2}{t}=2\left(2\right)=4\neq 0 \end{array} \end{array}$$

Since ${ϼ}_{*}\left(\dot{r},\dot{s}\right)={ϼ}_{+}\left(\dot{r},\dot{s}\right)\;{ϼ}_{-}\left(\dot{r},\dot{s}\right)$

$${ϼ}_{*}\left(\dot{r},\dot{s}\right)=\left(4\right)\;\left(4\right)=16\neq 0$$

But $\;{ϼ}_{\mathrm{LM}}\left(\dot{s},\dot{r}\right)=\sqrt{\left|{ϼ}_{*}\;\left(\dot{s},\dot{r}\right)\;{ϼ}_{*}\left(\dot{r},\dot{s}\right)\right|}$ = 0

Hence ṡ $\;{⊥}_{{ϼ}_{\mathrm{LM}}}\;\dot{r}\;$ but the others are not.

Example 3.4: Consider the real normed space $\dot{X}$ = $R^{2}$ with the norm $\left\|\dot{s}\right\|$ = $\left|{\dot{s}}_{1}\right|+\left|{\dot{s}}_{2}\right|$ where $\dot{s}=\left(1,0\right)$ ,

$\dot{r}=\left(0,1\right)$ we have $\;{ϼ}_{+}\left(\dot{s},\dot{r}\right)$ = $\left\|\dot{s}\right\|\;\lim_{t\;\to \;0^{+}}\frac{\left\|\dot{s}+t\dot{r}\right\|-\left\|\dot{s}\right\|}{t}$ and $\;{ϼ}_{-}\left(\dot{s},\dot{r}\right)$ = $\left\|\dot{s}\right\|\;\lim_{t\;\to \;0^{-}}\frac{\left\|\dot{s}+t\dot{r}\right\|-\left\|\dot{s}\right\|}{t}$

$\|\dot{s}\|=1,\|\dot{r}\|=1,$

$\left\|\dot{s}+t\dot{r}\|=\|(1,t)\|=|1|+|t\right|$

and $\left\|\dot{r}+t\dot{s}\|=\|(t,1)\|=|t|+|1\right|$

Thus $\;{ϼ}_{+}\left(\dot{s},\dot{r}\right)$ = (1) $\;\lim_{t\to 0^{+}}\frac{1+t-1}{t}$ = 1

${ϼ}_{-}\left(\dot{s},\dot{r}\right)\;$ = $\left(1\right)\;\lim_{t\to 0^{-}}\frac{1-t-1}{t}$ = -1

$\;{ϼ}_{+}\left(\dot{r},\dot{s}\right)$ = (1) $\;\lim_{t\to 0^{+}}\frac{t+1-1}{t}$ = 1

$\;{ϼ}_{-}\left(\dot{r},\dot{s}\right)\;$ = $\left(1\right)\;\lim_{t\to 0^{-}}\frac{-t+1-1}{t}\;$ = -1

We have $ϼ\left(\dot{s},\dot{r}\right)$ = $\frac{\;\;{ϼ}_{-}\left(\dot{s},\dot{r}\right)+{ϼ}_{+}\left(\dot{s},\dot{r}\right)\;}{2}$ therefore $\;ϼ\left(\dot{s},\dot{r}\right)$ = $\frac{-1+1}{2}$ = 0,

Since $\;{ϼ}_{\mathrm{gg}}=\sqrt{\left|\;ϼ\left(\dot{s},\dot{r}\right)\right|\;\left|\;ϼ\left(\dot{r},\dot{s}\right)\right|}\;$ implies ${ϼ}_{\mathrm{gg}}\left(\dot{s},\dot{r}\right)$ = $\sqrt{0}$ = 0,

Also ${ϼ}_{\lambda }\left(\dot{s},\dot{r}\right)$ = λ $\;{ϼ}_{-}\left(\dot{s},\dot{r}\right)$ + (1- λ) $\;{ϼ}_{+}\left(\dot{s},\dot{r}\right)$ ) = λ (−1) + (1- λ) (1)= 1-2λ put λ = $\frac{1}{2}\;$ then ${ϼ}_{\lambda }\left(\dot{s},\dot{r}\right)=0,$

But ${ϼ}_{\mathrm{LM}}\left(\dot{s},\dot{r}\right)\;$ = $\sqrt{\left|{ϼ}_{*}\left(\dot{s},\dot{r}\right)\;{ϼ}_{*}(\dot{r},\dot{s})\right|}\;$ = $\sqrt{\left|1\right|}$ = 1 ≠ 0

Hence $\;\;{⊥}_{ϼ}$ ⊈ $\;{⊥}_{{ϼ}_{\mathrm{LM}}}$ , $\;\;{⊥}_{{ϼ}_{\mathrm{gg}}}⊈\;{⊥}_{{ϼ}_{\mathrm{LM}}}$ and $\;\;{⊥}_{{ϼ}_{\lambda }}⊈$ $\;{⊥}_{{ϼ}_{\mathrm{LM}}}.$

Example 3.5: Consider the real normed space $\dot{X}$ = $R^{2}$ with the norm $\left\|(\dot{s},\dot{r}\;)\right\|$ = max { $\left|\dot{s}\right|,\left|\dot{r}\right|$ } where $\dot{s}=\left(1,1\right)$ , $\dot{r}=\left(0,-1\right)$ . Then for every $\dot{s},\dot{r}\;\in \;\dot{X}$ we have

$$\begin{array}{c} {ϼ}_{+}\left(\dot{s},\dot{r}\right)=\left(1\right)\;\lim_{t\;\to \;0^{+}}\frac{\left|1\right|-1}{t}=1\left(0\right)=0\\ {ϼ}_{-}\left(\dot{s},\dot{r}\right)=\left(1\right)\lim_{t\;\to \;0^{-}}\frac{\left|1-t\right|-1}{t}=1\;\left(-1\right)=-1\\ \begin{array}{c} {ϼ}_{+}\left(\dot{r},\dot{s}\right)=\left(1\right)\;\lim_{t\;\to \;0^{+}}\frac{\left|t\right|-1}{t}=1\left(0\right)=0\\ {ϼ}_{-}\left(\dot{r},\dot{s}\right)=\left(1\right)\;\lim_{t\;\to \;0^{-}}\frac{\left|-1+t\right|-1}{t}=\lim_{t\;\to \;0^{-}}\frac{\left|t-1\right|-1}{t}=-1 \end{array} \end{array}$$

We have $ϼ\left(\dot{s},\dot{r}\right)$ = $\frac{\;\;{ϼ}_{-}\left(\dot{s},\dot{r}\right)+{ϼ}_{+}\left(\dot{s},\dot{r}\right)\;}{2}$ therefore $ϼ\left(\dot{s},\dot{r}\right)$ = $\frac{-1+0}{2}$ = $\frac{-1}{2}$ ≠ 0 and $ϼ\left(\dot{r},\dot{s}\right)$ = $\frac{\;\;{ϼ}_{-}\left(\dot{r},\dot{s}\right)+{ϼ}_{+}\left(\dot{r},\dot{s}\right)\;}{2}$ = $\;\frac{-1+0}{2}$ = $\frac{-1}{2}\;$

$${ϼ}_{\mathrm{gg}}\left(\dot{s},\dot{r}\right)=\sqrt{\left(\frac{-1}{2}\right)\left(\frac{-1}{2}\right)}=\sqrt{\left(\frac{1}{4}\right)}\;=\frac{1}{2}\neq 0$$

$${ϼ}_{\lambda }\left(ṡ,\dot{r}\right)=\lambda \;\;{ϼ}_{-}\left(\dot{s},\dot{r}\right)+\left(1-\lambda \right)\;\;{ϼ}_{+}\left(\dot{s},\dot{r}\right))=\lambda \left(-1\right)+\left(1-\lambda \right)\;\left(0\right)=-\lambda \neq 0$$

but ${ϼ}_{\mathrm{LM}}$ (ṡ, $\;\dot{r}$ ) = $\sqrt{\left|{ϼ}_{*}\left(\dot{s},\dot{r}\right)\;{ϼ}_{*}(\dot{r},\dot{s})\right|}\;$ = 0.

Hence $\;{⊥}_{{ϼ}_{\mathrm{LM}}}$ ⊈ $\;{⊥}_{ϼ}$ , $\;\;{⊥}_{{ϼ}_{\mathrm{LM}}}⊈\;{⊥}_{{ϼ}_{\mathrm{gg}}}$ and $\;{⊥}_{{ϼ}_{\mathrm{LM}}}⊈$ $\;{⊥}_{{ϼ}_{\lambda }}\;$

Remark 3.6: From example 3.4 and example 3.5 it follows that the orthogonalities, $\;{⊥}_{ϼ}$ , $\;{⊥}_{{ϼ}_{\mathrm{gg}}}$ , $\;{⊥}_{{ϼ}_{\lambda }}$ and $\;{⊥}_{{ϼ}_{\mathrm{LM}}}$ are generally incomparable.

Remark 3.7: If *X* is smooth then $\;\;{⊥}_{{ϼ}_{-}}=\;{⊥}_{{ϼ}_{+}}=\;{⊥}_{ϼ}=\;{⊥}_{{ϼ}_{*}}=\;{⊥}_{{ϼ}_{\lambda }}$ but not equal to $\;{⊥}_{{ϼ}_{\mathrm{LM}}}$ .

## 4. Conclusion

This paper presented a new orthogonality relation in normed spaces, termed ${ϼ}_{\mathrm{LM}}$ -orthogonality, introduced via ${ϼ}_{*}$ -orthogonality. Several fundamental properties were established, among them symmetry, non-negativity, homogeneity, and a Cauchy–Schwarz type inequality.

The connections between ${ϼ}_{\mathrm{LM}}$ -orthogonality and other classical orthogonality concepts were examined, revealing that these notions are generally non-equivalent. Collectively, the results confirm that ${ϼ}_{\mathrm{LM}}$ -orthogonality constitutes a meaningful and structurally rich extension of existing frameworks.

Possible directions for future work include characterizing linear mappings that preserve ${ϼ}_{\mathrm{LM}}$ -orthogonality and investigating its role in the geometry of normed spaces.

### Ethical considerations

This study is entirely theoretical and does not involve any human participants or animals, nor does it rely on data requiring ethical approval. Therefore, no ethical concerns arise in conducting this research.

## Data Availability

This study is entirely theoretical and does not rely on any experimental or external datasets. Therefore, no data are available in connection with this research. This article is a purely theoretical study in functional analysis and does not involve clinical trials, animal studies, observational studies, or qualitative research. Therefore, standard reporting guidelines such as CONSORT, ARRIVE, STROBE, or COREQ/SRQR do not directly apply. Standard practices for presenting theoretical mathematical results have been followed, including clear definitions, theorems, proofs, and logical consistency.
