# Supplementary material for: New Kind of orthogonality in Normed spaces by using ϼ * -orthogonality
Source: F1000Res. 2026 May 27;14:1486. Originally published 2025 Dec 31. [Version 2] doi: 10.12688/f1000research.174190.2 (PMC13184956; doi:10.12688/f1000research.174190.2)
Supplement: Peer review history

# Reviewer report: Reviewer response for version 2

**DOI:** 10.5256/f1000research.201643.r488721

**Published:** 2026-06-10

**Authors:** Umar Ishtiaq

**Affiliations:** 1 University of Management and Technology, Lahore, Pakistan

The authors revised the manuscript according to my suggestions.

Is the work clearly and accurately presented and does it cite the current literature?

No

If applicable, is the statistical analysis and its interpretation appropriate?

Not applicable

Are all the source data underlying the results available to ensure full reproducibility?

Yes

Is the study design appropriate and is the work technically sound?

Yes

Are the conclusions drawn adequately supported by the results?

No

Are sufficient details of methods and analysis provided to allow replication by others?

Yes

Reviewer Expertise:

Functional Analysis

I confirm that I have read this submission and believe that I have an appropriate level of expertise to confirm that it is of an acceptable scientific standard.

# Reviewer report: Reviewer response for version 1

**DOI:** 10.5256/f1000research.192072.r477500

**Published:** 2026-05-14

**Authors:** Arul Joseph Gnanaprakasam

**Affiliations:** 1 SRM Institute of Science and Technology, Kattankulathur, Tamil Nadu, India

The authors introduced a new notion of orthogonality based on norm derivatives and established several interesting properties related to this concept. The proposed orthogonality is discussed in comparison with existing notions in the literature, and the paper provides useful remarks and illustrative examples to clarify the results.

 The manuscript is mathematically sound and well organized. The topic is relevant to current research in normed spaces and orthogonality theory, and the obtained results may motivate further studies in this direction. Overall, the paper makes a valuable contribution to the subject and is suitable for indexingin its present form.

Is the work clearly and accurately presented and does it cite the current literature?

Yes

If applicable, is the statistical analysis and its interpretation appropriate?

Partly

Are all the source data underlying the results available to ensure full reproducibility?

Yes

Is the study design appropriate and is the work technically sound?

Yes

Are the conclusions drawn adequately supported by the results?

Yes

Are sufficient details of methods and analysis provided to allow replication by others?

Partly

Reviewer Expertise:

Functional Analysis

I confirm that I have read this submission and believe that I have an appropriate level of expertise to confirm that it is of an acceptable scientific standard.

# Reviewer report: Reviewer response for version 1

**DOI:** 10.5256/f1000research.192072.r468033

**Published:** 2026-04-30

**Authors:** Souvik Ghosh

**Affiliations:** 1 Jadavpur University, Kolkata, West Bengal, India

The authors tried to provide a new orthogonality based on norm derivatives. The orthogonality here mentioned is coarser than other orthogonalities in the literature. Although they showed that it is incomparable with some other orthogonalities followed by some remarks. The authors may look for the following problems for further exploration. For instance by investigating the quantitative invariant other gap between two different (incomparable) orthogonalities. Also, there is a scope to study the preservation of such orthogonality.

Is the work clearly and accurately presented and does it cite the current literature?

No

If applicable, is the statistical analysis and its interpretation appropriate?

Not applicable

Are all the source data underlying the results available to ensure full reproducibility?

Yes

Is the study design appropriate and is the work technically sound?

Partly

Are the conclusions drawn adequately supported by the results?

Yes

Are sufficient details of methods and analysis provided to allow replication by others?

No

Reviewer Expertise:

Geometry of Banach spaces (Functional Analysis)

I confirm that I have read this submission and believe that I have an appropriate level of expertise to confirm that it is of an acceptable scientific standard.

# Reviewer report: Reviewer response for version 1

**DOI:** 10.5256/f1000research.192072.r458032

**Published:** 2026-03-19

**Authors:** Umar Ishtiaq

**Affiliations:** 1 University of Management and Technology, Lahore, Pakistan

The authors need to address the following issues:

1. The manuscript contains several typographical errors and formatting inconsistencies, such as repeated sentences in the introduction and missing mathematical symbols in Theorem 1.1.
2. The definition of $\rho_{LM}$-orthogonality in Definition 2.1 is clear, but the motivation for introducing this specific form of orthogonality could be better justified.
3. The proof of Proposition 2.2 contains multiple formatting issues and missing parentheses, making some steps difficult to follow.
4. In Example 3.4, the calculation of $\rho_{LM}(\dot{s},\dot{t})$ appears to contain an error, as the result should be verified more carefully.
5. The references are inconsistently formatted, with some entries missing page numbers or having incomplete information (e.g., references 4, 5, and 6).
6. The paper references figures and tables that are not included in the provided PDF, making it difficult to verify the claims made in examples.
7. The paper lacks a formal conclusion section to summarize the main findings and their significance in the context of existing orthogonality concepts.

Is the work clearly and accurately presented and does it cite the current literature?

No

If applicable, is the statistical analysis and its interpretation appropriate?

Not applicable

Are all the source data underlying the results available to ensure full reproducibility?

Yes

Is the study design appropriate and is the work technically sound?

Yes

Are the conclusions drawn adequately supported by the results?

No

Are sufficient details of methods and analysis provided to allow replication by others?

Yes

Reviewer Expertise:

Functional Analysis

I confirm that I have read this submission and believe that I have an appropriate level of expertise to confirm that it is of an acceptable scientific standard, however I have significant reservations, as outlined above.
